# An analysis of the role of different levels of exchange of explicit information in human–robot cooperation

**DOI:** 10.3389/frobt.2025.1511619

**Published:** 2025-02-10

**Authors:** Ane San Martin, Johan Kildal, Elena Lazkano

**Affiliations:** ^1^ Department of Autonomous and Intelligent Systems, Tekniker, Eibar, Spain; ^2^ Faculty of Informatics, University of the Basque Country (UPV/EHU), Bilbao, Spain

**Keywords:** human–robot collaboration, bidirectional communication, situation awareness, social signal processing, user experience, user study

## Abstract

For smooth human–robot cooperation, it is crucial that robots understand social cues from humans and respond accordingly. Contextual information provides the human partner with real-time insights into how the robot interprets social cues and what action decisions it makes as a result. We propose and implement a novel design for a human–robot cooperation framework that uses augmented reality and user gaze to enable bidirectional communication. Through this framework, the robot can recognize the objects in the scene that the human is looking at and infer the human’s intentions within the context of the cooperative task. We proposed three levels of exchange of explicit information designs, each providing increasingly more information. These designs enable the robot to offer contextual information about what user actions it has identified and how it intends to respond, which is in line with the goal of cooperation. We report a user study (n = 24) in which we analyzed the performance and user experience with the three different levels of exchange of explicit information. Results indicate that users preferred an intermediate level of exchange of information, in which users knew how the robot was interpreting their intentions, but where the robot was autonomous to take unsupervised action in response to gaze input from the user, needing a less informative action from the human’s side.

## 1 Introduction

In Industry 4.0, the manufacturing sector has advanced in developing smarter and more adaptable systems (Fechter et al., 2016; Michalos et al., 2010), which have fueled increased adoption of cobots, in spaces shared with humans (Grau et al., 2020). The adaptability of a robot to the needs of its human peer is key for obtaining an effective collaboration between humans and robots (Nikolaidis et al., 2017; Buerkle et al., 2022). The robot’s ability to understand, interpret, and respond to the diverse requirements and preferences of the user is important to ensure seamless interaction and cooperation. By adapting to the users’ needs, robots can personalize their responses, actions, and behaviors, thereby enhancing communication and productivity. A robot that adapts to the user fosters a collaborative environment where both humans and machines work synergistically, thus optimizing efficiency and achieving mutual goals while accommodating the unique needs of each user.

To provide robots with the capability to adapt to user needs, social cues play an important role (Fiorini et al., 2023; Kansizoglou et al., 2022), facilitating effective communication, comprehension, and cooperation between humans and machines. Communication between people is characterized by the use of a range of different nonverbal cues (Argyle, 1972; Mandal, 2014). These cues encompass a wide array of nonverbal signals, including facial expressions, gestures, body language, tone of voice, and contextual cues (Burgoon et al., 2011). Enabling these same communication channels between humans and robots or other interactive agents provides the opportunity for humans to obtain more natural, inviting, and accessible interactive experiences (Alves et al., 2022; Terzioğlu et al., 2020; Romat et al., 2016). As an example, being able to track the gaze of the human can be a powerful tool for it to understand and identify user intentions in human–robot interactions (HRIs) (He et al., 2018; Singh et al., 2020; Admoni and Scassellati, 2017). By tracking and analyzing a user’s eye movements and gaze direction, robots can infer valuable insights about the user’s intentions, interests, and focus. This enables robots to adjust their behavior, orient themselves toward the user’s gaze, and even anticipate the user’s needs, thereby enhancing the overall interaction and collaboration between humans and machines (Kompatsiari et al., 2017; Saran et al., 2018).

While social cues can enhance interactions during collaboration with robots, ensuring a good user experience (UX) involves considering various other factors, among which situation awareness (SA) holds an important role (van de Merwe et al., 2022; Ososky et al., 2012). Situation awareness refers to the user’s understanding of the ongoing situation or context. In human–robot collaboration, it is important to provide the user with contextual information so that they can understand what is happening while they are collaborating with robots. According to Endsley (1995), SA is a hierarchical construct consisting of three levels:

*Level 1:* this level involves recognizing and identifying key elements in the environment that are crucial to the task at hand. In human–robot cooperation (HRC), this may include perceiving the robot’s actions, its current state, or the status of the shared task.

*Level 2:* In this level, the user integrates and interprets the perceived information to form a coherent understanding of the current state. For instance, the user might understand how the robot’s actions contribute to the collaborative goal.

*Level 3:* The final level involves using the current understanding to anticipate what will happen next. In HRC, this could mean predicting the robot’s next steps or the evolution of the collaborative task.

All these levels of SA ensure that users do not only perceive relevant information but also understand its importance and anticipate future steps. This hierarchy highlights the dynamic and evolving nature of SA. However, it is important to recognize that in interactions between agents, users do not need to understand the surrounding with absolute accuracy. Instead, successful interactions rely on humans’ ability to make and continuously update assumptions and attributions about the robot’s intentions based on key evidence (Thellman et al., 2022; Thellaman et al., 2017). Hence, providing users with enough information to identify the robot’s intentions, aligned with the goals of the interaction, is enough for effective collaboration. Enhancing contextual information in this way directly contributes to the user’s cognitive understanding and task success (Riley et al., 2012). In this context, the use of mixed reality (MR) techniques in HRI has increased, showing promising results in delivering better information (Bagchi and Marvel, 2018; Green et al., 2007; Pan et al., 2012; Rowen et al., 2019).

Adding to this line of research, the main contributions of this paper are as follows:

(i) Development of a bidirectional communication framework using eye-tracking on HoloLens: we report all the steps to be followed and technology needed to obtain a bidirectional communication in HoloLens for HRC tasks in industry.
(ii) Design and implementation of different explicit information levels: we introduce and implement three levels of exchange of explicit information MR displays to evaluate the impact of the extent of contextual information provided to users in HRC scenarios.
(iii) Empirical evaluation of the information displays: this work analyzes the impact of the three different MR displays on UX and performance by reporting a user study (n = 24), where users performed a cooperative task with a robot. The cooperative task required the robot and the human to understand each other to cooperatively construct a building using blocks from a building set, with some of the blocks placed on the human side and others on the robot’s side.

The rest of the paper is structured as follows. We first analyze the current state of the art in gaze tracking, non-verbal communication, and the use of MR in HRI to enhance contextual information and hence UX. After the related work, we describe and discuss how the robot tracks the user’s gaze in order to identify which object in the scene does the user want. Then, we describe the three different MR displays developed, in which a different level of exchange of explicit information is used. We also report an experimental user study (n = 24) that compares the performance and subjective experience obtained from each display developed, while completing a task in collaboration with a robot in a space that is shared with a moving physical robot. Finally, we analyze the results obtained and draw conclusions from them.

## 2 Related work

In recent years, with Industry 4.0 as the background, a strand of the research conducted in HRI has focused on investigating how robots can best adapt to the needs of users, shifting focus from robots toward humans (Longo et al., 2022; Jakl et al., 2018; Brauner et al., 2022). Robots must understand humans to some extent if they are expected to adapt to them and provide the means for a good exchange of information. To that end, it is essential to know how humans understand each other.

Humans are able to identify the other person’s state by decoding information transmitted through non-verbal communication channels, reducing the need for verbal communication and, hence, significantly minimizing delays in state understanding (Harrison, 1989; Tracy et al., 2015). For a robot to understand a human, it must evaluate the internal states of users, primarily focusing on their intended objectives and, to some degree, their motivations and emotions (Breazeal et al., 2005; Romat et al., 2016; Weidemann and Rußwinkel, 2021). Among humans, this understanding is established through an ongoing exchange of implicit signals conveyed through behavior. Examples of these signals are facial expressions (Rawal and Stock-Homburg, 2022; Hu et al., 2024; Deng et al., 2019) and body language (Romeo et al., 2021; Martinez et al., 2016), both of which can convey valuable information about the user’s status, and eye gaze (Frischen et al., 2007; Bader et al., 2009), which can also provide information about the focus and intention of users. Focusing on the importance of eye gaze, some researchers have carried out exploration by utilizing this information to enhance collaboration between humans and robots. Shi et al. (2021) proposed a new algorithm, which improved the accuracy of the identification of the objects that the user wants for a human–robot collaboration scenario. In this line, Yang et al. (2023) proposed a new method to more accurately identify the object that the user wanted to improve the intention recognition of users with the aim of obtaining a more natural grasp intention in an HRI environment. Similarly, Fuchs and Belardinelli (2021) analyzed how eye gaze could be used to predict intention while tele-operating a robotic gripper during a simulation. This approach investigated eye–hand coordination and developed an intention estimation model for real-world shared autonomy. Additionally, Zheng et al. (2021) also analyzed how eye gaze information could be used to identify the user’s motion, which is a critical aspect to be considered in assistive robotics.

However, having a robot that can adapt to the user’s needs is not enough to obtain a good UX. This can be overwhelming and annoying to users because they may feel a lack of control (Norman, 1994; Maier et al., 2006). In recent years, MR techniques have been proven to be useful to address this issue as they are able to provide good contextual information and, hence, reducing anxiety and improving performance while collaborating with robots (Brending et al., 2016). Szafir (2019) explored the integration of virtual, augmented, and MR with robotics. They analyzed that such technologies improve mediation between humans and robots, enhancing interaction. Their pioneering use of modern virtual, augmented, and MR hardware showed improvements in both objective performance and UX, where they aimed to augment shared environments, robots, and user interfaces—an invitation to unite diverse fields. Chen et al. (2023) showed how using augmented reality (AR) in human–robot collaboration for construction waste sorting can lead to safe and productive environments and improve the accuracy in by 10%–15%. De Franco et al. (2019) presented an AR interface used in HRC tasks, which enabled real-time communication between the user and the robot, introducing performance benefits. Their results showed the effectiveness of their AR interface and suggested that the subjects were satisfied with the task carried out with the AR feedback. Lastly, San Martin et al. (2023) examined the role of audio, visual, and audio-visual displays in a safety context, particularly in shared-space human–robot collaboration scenarios. They concluded that all types of displays were suitable for safe human–robot collaboration scenarios. However, qualitative differences were observed, indicating that each type of display could contribute differently to users’ perception and experience.

Nevertheless, these techniques do not analyze the effect of adding bidirectional communication and the benefits it can provide in collaborative scenarios. In this line, Chadalavada et al. (2020) examined how human eye gaze could convey navigation intent to robots, recommending the use of eye-tracking glasses in industrial settings to improve human-to-robot communication and predictability and combining it with AR to provide information of the trajectory of the mobile robot. In a similar way, Lee et al. (2023) introduced a novel approach for controlling a mobile robot using a head-mounted device (HMD). This method leverages the HMD to display the robot controller and capture human eye-gaze within an AR environment, enabling hands-free control for enhanced multitasking and efficiency in human–robot collaboration. Comparative experiments with joystick control indicated that hands-free control using the HMD provided precise and effective robotic operation, presenting a viable alternative to traditional joystick-based approaches. Following the hand-free controlling idea, Park et al. (2021) introduced a hands-free HRI method in MR using eye gazing and head gestures with deep learning for intuitive robot control. This approach, including object-based indirect manipulation and a digital twin for simulation, was shown to be more efficient and effective than traditional direct interaction methods. In contrast to the hand-free control area, Weber et al. (2022) presented a method for flexible HRI in different environments using AR and eye tracking. The approach enabled fast, user-friendly calibration of robot sensors for 3D detection and localization of unknown objects without pre-training. By integrating the HMD, the method provided visual feedback in AR, thus facilitating intuitive interactions and actions such as object grasping.

Although advancements in MR technologies have increased their usage in human–robot collaboration applications, showing their potential to provide good information, it must still be analyzed as to how important is the role of the amount of information exchanged between humans and robots. The proposed MR solutions for human–robot collaboration and cooperation often analyze the effects of providing or not providing information or even the computation between different technologies; however, they do not analyze the effects of using different levels of exchange of explicit information. Our application is based on the development of three different levels of contextual information, combining both audio and visual information, while users are working together with a robot. Depending on the level used, the application will vary on the amount of information displayed and on the information required from users. The system always provides cues for the user to check whether the system is performing item identifications correctly. In the highest level of information, it even explicitly informs if the users want the robot to start moving, requiring from users more information than in other levels. Concurrently, our work aims to evaluate and assess the effectiveness and UX experienced in each different level and analyze how the performance and personal experience are affected depending on the amount of information exchanged between the robot and human user. This contributes to developing an understanding on how the amount of information exchanged, through multimodal bidirectional communication, can either improve or worsen HRC scenarios.

## 3 Developing a bidirectional communication

Identifying user intents or desires will be a key in the development of bidirectional communication with the robot. Eye gaze can offer insights into what the user is focusing on, thereby making it possible to identify what the user wants within the context of the interaction. However, a two-way communication is required for good collaboration. The user should also be able to interpret the robot’s “mind” and thus be aware of the situation with respect to the robot in the context of the collaboration. With that goal in mind, we propose displaying information to the user regarding the cooperation with the robot.

In the scope of this paper, the HRC scenario involves a worker sharing space with an LBR iiwa 14 robot, where both the robot and human cooperate to complete a task. Specifically, the robot and the human have to understand each other in order to construct a building together using blocks from a building set.

**FIGURE 1:**
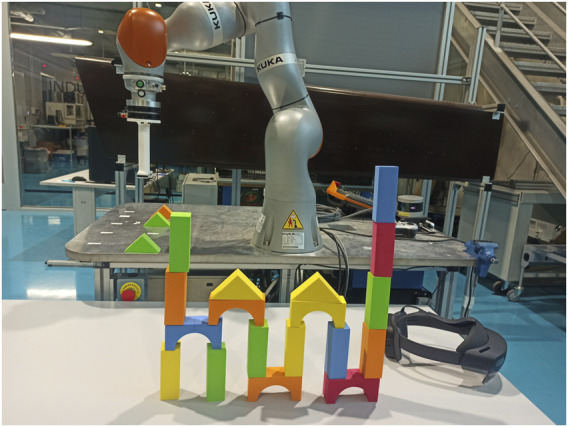
This image shows a view of the cooperative scenario where users and a robot worked together to construct a building using building blocks. One of the buildings created by a user is displayed in the image. The user was seated in front of the table, from where the image was taken. There was set of pillar (rectangle) building blocks on this table. Bridges and triangular blocks were located on the robot’s table, where the robot was positioned. When the user needed any of these pieces, the robot would move closer to them. For bidirectional communication, users used the HoloLens.

The user will have pillars that can be directly used for the construction. The robot owns the bridges and triangles, which the users will have to request from the robot in order to use them in the building process. Figure 1 shows an example of one of the buildings built by a user in cooperation with the robot.

The user will wear a HoloLens 2 HMD device for eye tracking, visualization of different levels of explicit information, and for interacting with the robot. The communication between both sides (user and robot) is fulfilled through the use of ROS-TCP and ROS services through the use of two computers (one server and the robot’s computer).

For the implementation, we used Unity (v.2020.3.11f1) and the Unity Robotics Hub (V.0.7.0) with ROS-TCP Connector (v.0.7.0) in order to connect with the Robot Operating System (ROS). Two Dell computers with an Intel CPU i7 and 32 GB RAM were used. The first computer (server computer) established the connection with HoloLens using WebRTC (v.2.0.2)^1^. Yolo v5^2^ NN was also running in this computer in order to identify objects in the image coming from the HoloLens, and it would also connect to the second computer, built in the robot, by the use of ROS. In the second computer, MoveIt^3^, was used in order to make the robot move to any desired target position.

### 3.1 How to identify what the user wants

In gaze-based HRI, fixation is often used as an indication of the visual intention of a human. Tracking the user’s gaze involves analyzing pupil images and mapping them onto the scene image, thus providing insights into a person’s visual intent. Various eye movements characterize gaze, with fixation and saccade being among the most prevalent types. Fixation is characterized by a stable gaze within a confined area, while saccade denotes swift eye movements (Holmqvist et al., 2011). Fixation can be from as short as 50 ms to several seconds in duration (Velichkovsky et al., 2019; Liu, 2014). In the context of HRC, fixation time must be carefully set up since an inappropriate short value can lead to accidentally wrong selections, and large values can eventually produce eye fatigue and inefficiencies. The HoloLens 2 device is capable of gathering gaze data at 30 fps^4^, and this means that we have information of the gaze position every 33 ms. However, since gaze data were sent to the main computer for object identification, this could introduce delays in the whole process. After measuring and analyzing, we deduced a mean response time of 235 ms. Additionally, we conducted several tests to identify the time required for the system to consistently identify the same piece, ensuring it reflected that the user wanted the piece and not just glancing over. We identified that 600 ms fixation time worked well for our approach, corresponding to three consecutive gaze data points on the same object. To send the gaze data to the server, the eye gaze data were transformed from the 3D world to 2D screen position (pixel value). Streaming of images and data of the eye gaze relied on Web Real-Time Communication (WebRTC) protocol, using MixedReality-WebRTC library, as reported by Barz et al. (2021). With each gaze update, the frame and gaze data were streamed to the server, which would identify and return the object that the user was looking at, if any.

### 3.2 Identifying the desired object and identifying the object placed

When the gaze data and image arrived at the server, the next step was identifying if the user was looking at any object. For this step, we decided using You Only Look Once v5 (YOLO v5) neural network (NN), which has been widely used for object detection tasks (Kim et al., 2022; Sirisha et al., 2023; Yu et al., 2022). To adapt the NN to our scenario, in order to detect the objects of interest (pillars, bridges, and triangles), we recorded videos performing the task with HoloLens 2 used in the study. We recorded videos of a total duration of 1 h 17 min, from where we labeled a total of 1,540 images, which were augmented up to three times, with tools provided by Roboflow^5^. Roboflow was also used for the labeling of the data, where 75% of the data were used for training, 17% were used for validation, and the remaining 8% for the testing. We first used the *–evolve* parameter to identify the optimal set of hyperparameters for training. We used 300 iterations to identify the best-performing hyperparameters, which were then used to train the model with a batch-size value of 8 and 400 epochs.

To evaluate the model’s performance, we analyzed two metrics obtained from the training of the model: F1 score and mean average precision (mAP), specifically mAP@0.5:0.95. The F1 score was used to evaluate the performance of the model. The F1 score evaluates model performance by balancing precision (correct positive predictions out of all predicted positives) and recall (correct positive predictions out of all actual positives). It combines both precision and recall into a single value in a range [0–1], where 1 indicates perfect precision and recall. The mAP metric is commonly used in object detection tasks. It provides a detailed evaluation of object detection performance across a range of Intersection over Union (IoU) thresholds, offering insights into both localization accuracy and detection confidence. IoU quantifies the extent of overlap between the predicted bounding boxes and the actual ground truth bounding boxes. Within the mAP metric, average precision is computed across a range of IoU thresholds spanning from 0.5 to 0.95. This approach offers a holistic assessment, encompassing not only localization accuracy but also the model’s confidence in detecting objects. From the model trained, we obtained an F1 score of 0.7209 and an mAP@0.5:0.95 of 0.69667.

**FIGURE 2:**
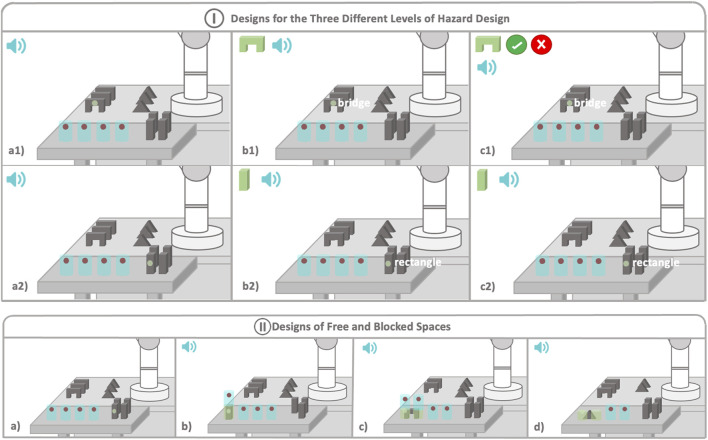
This image provides a schematic view of the cooperative scenario, illustrating (I) the three different designs implemented and (II) explaining what occurred each time a piece was blocked in a desired space. I. Design of *Three Different Levels of Explicit Information*. In all three cases, blue cylinders represent the free spaces where users can place the objects. Figures (a1) and (a2) illustrate the designs implemented for the C1 condition, where only audio display is utilized to notify the user about the system’s good performance. Figures (b1) and (b2) showcase the type of information designed for the C2 condition, incorporating both audio and visual elements. Figures (c1) and (c2) depict the design implemented for the C3 condition. In this case, the design for the pillars remains consistent with C2, but it varies for the pieces held by the robot, where the user is prompted to confirm the identification. The speaker signal means that a sound is played when an object is identified. II. Design of *Free and Blocked Spaces*. In all three cases, blue cylinders represent the free spaces where users can place the objects. Users will look at the red point of the desired free space to block it. Figure (a) represents the free spaces where users can place objects. Figure (b) shows what happens when a pillar is blocked in a space. Figure (c) shows the placement of a bridge, and (d) shows placement of a triangle. The speaker signal means that a sound is played when free space is blocked.

Once the model was trained and validated, it was used in the first computer (the server) to identify objects in the frames. Every time new data arrived at the server, it would process the image and identify all the objects in the image by creating bounding boxes around them. We also created a script to identify whether the gaze coordinates fell within any bounding box. If they did, the name of the object was sent back to the HoloLens. In HoloLens, it was analyzed if the user had been staring at the same object for more than the stipulated time (600 ms, see Section 3.1). If this happened, in all cases, a sound was played (see Figure 2 (I)) to make users aware that the object had been identified and they could proceed to pick the piece up and place it in the desired free space. Free spaces were represented as holographic blue cylinders with red spheres (see Figure 2). In C2 and C3, not only was a sound played but the corresponding hologram was also displayed (see Figure 2 (I)). It has to be noted that the system was capable of identifying changes in the user’s desired object. When the user shifted their attention to another object, the system would update this information by again playing the sound and in the corresponding case, displaying the identified new hologram.

The identification of objects was also used when an object was placed. Users could place an object by looking at the red sphere of the desired place for 2 s. The system would then identify if the gaze was colliding with the sphere and whether during the same period of time, the gaze lied inside the object identified. When the space was locked, the system would no longer identify this object, even when the user looked at it, since the system identified it as a placed object. To achieve this, we added a cylindrical collider in the same place of the blocked space so that if the gaze collided with it, the system would skip identifying the object.

### 3.3 Designing the three different levels of exchange of explicit information

For the user to obtain good information aligned with the goal of the task, we focused on the following three aspects of the information presented to users: *what*, *how much*, and *how* the information is presented to users.

Regarding the information provided to users (*what*), as suggested by Thellman et al. (2017) and Thellman et al. (2022), users do not need whole-context information to have a good interaction with robots; instead, they need proper information to be able to continuously update the robot’s intentions. Hence, we provide users with updates about the robot’s state and intentions. Concerning *how much* information we provided, we designed three different levels of exchange of explicit information, where the amount of information provided to users varied based on the chosen level. Finally, regarding the manner (*how*) in which information was presented, as presented by San Martin et al. (2023), audio displays are effective in capturing users’ attention, while visual displays provide understandable information about the system. Therefore, we used both types of displays to provide information to the users.

Having these aspects in mind, we designed interactions with three incremental levels of amounts of explicit information exchanged between the robot and the human:

#### 3.3.1 Low level of exchange of explicit information (C1)

In this level, the smallest amount of explicit information was exchanged between the robot and user. Robots only received gaze information from users, and users were provided with the minimum information they needed to be sure that the robot was understanding and working. When the system identified that a user wanted an object, a confirmation sound was played, which indicated that the robot had identified the object. Once identified, the user could pick up the piece and place it in any of the free spaces shown by the HMD, which were displayed to the users with blue cylindrical holograms (see Figure 2 (I)). If the object identified was any of the objects placed on the robot’s side, then the robot pushed the object closer to the user (see Figure 2 (I), a1)).

#### 3.3.2 Middle level of exchange of explicit information (C2)

In this second case, the robot was again provided with only gaze information from the user. However, in this level, users received the essential information explained in C1 along with further explicit information. In this level, the system displayed, at real-time, the name of the object the user was looking at, and when the system identified that the user wanted an object, it not only played a sound but also displayed a hologram of the identified piece in the left corner of the HoloLens device (see Figure 2 (I), b1) and b2)). As in the previous case, the user could then place the piece in any free space or wait until the robot first moved the piece closer to them.

#### 3.3.3 High level of exchange of explicit information (C3)

The highest amount of explicit information is exchanged in this case. Robots did not only receive gaze information from users, but they also received confirmatory information, providing users with a greater control over the situation. In this case, everything worked in the same way as in C2 (see Figure 2 (I), c2)), unless when the user wanted a piece belonging to the robot. In this case, the HoloLens would not only play a sound and display the correspondent hologram but would also display an *accept* or *reject* hologram (see Figure 2 (I), c1)), asking the user whether the piece had been identified correctly, before bringing it to the user. The robot would not move until the user selected the *accept* button, by looking at it for 2 s. In case the *reject* button was selected, then the robot would not move.

In all three conditions, the user’s gaze was displayed by the HMD by showing a green pointer (see the green point in any of the images of Figure 2).

The placement and free space designs were the same for the three conditions. Free spaces were displayed by blue holographic cylinders with a red point in each one (see Figure 2 (II), a)). The pillars occupied just one place, and they were blocked by looking at the red point of the desired free space. Once the position was blocked, the red point disappeared, the cylinder turned green, a sound was played, and a new free space appeared in the upper side (see Figure 2 (II), b)). However, the bridges and triangles occupied two spaces, and the user had to look at the left space in order to block both spaces (see Figure 2 (II), c) and d)). The process of blocking the spaces was the same as with pillars, but in this case, the height was also reduced by half to fit the objects’ height. In the case of a bridge, two new spaces would appear in the upper side (see Figure 2 (II), c)). In case of the triangles, no more spaces appeared over it since we decided that this would be the top piece (see Figure 2 (II), d)). In the C2 and C3 conditions, when a place was blocked, the hologram of the identified element also disappeared.

### 3.4 HoloLens–robot communication

The last aspect in the implementation of the system corresponded to the robot’s motion. In the C1 and C2 designs, whenever the system identified a bridge or a triangle, the HoloLens would directly send information about which object the robot should move to the server. In the case of C3, this information would be sent after the user’s acceptance (by looking at the accept button for 2 s). Once the server identified which object the robot needed to move, it would be responsible for calling the corresponding ROS service, together with the name of the piece as an argument.

**FIGURE 3:**
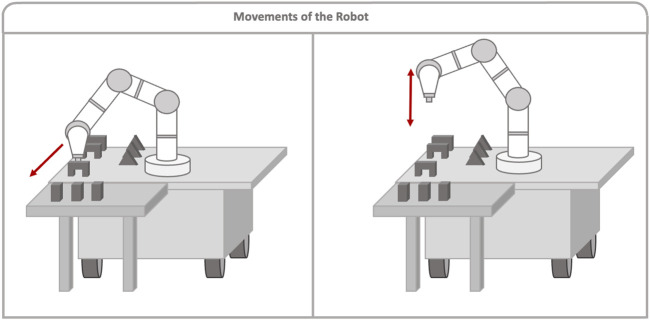
*Left image* shows the robot moving a piece close to the user when there is no such piece close to the user. In that case, the robot moves the nearest piece closer to the user so the user can take it. *Right image* shows the behavior of the robot when there is already a desired piece close to the user. In this case, the robot will only move down and up next to the piece.

A script was run on the robot´s computer, facilitating communication with the server computer and controlling the robot’s movement. This script also tracked how many pieces of each type were moved. In this way, each time the robot received information of the piece to move, it would know where to go and move the piece closer to the user’s reach so that the user can construct a building, as shown in Figure 3, left. The trajectories were planned and executed using the MoveIt library. As mentioned earlier, the system was also capable of identifying when the user changed their mind and wanted a different piece. For this reason, each time a piece was placed, this information was sent from HoloLens to the server, and the server would then call the corresponding ROS service so that the robot could keep track of the pieces placed. In this way, for example, if the user initially requested a bridge but then changed their mind and placed a pillar instead, the robot could track the already moved piece that was not used. Thus, if the user requested a bridge again, the robot would not move a new piece, but it would instead indicate the piece that had already been moved to the user, as shown in Figure 3, right.

## 4 User study

We conducted an experimental user study (n = 24), in which we compared the three interaction designs. To ensure participants’ safety, any sharp or pointy object that could harm the participants was removed from the scene. The robot was set to move slowly during the study, with a mean speed of 0.25 m/s, so that users could react easily to the robot’s movement. At the same time, the experimental study was performed in an open space where participants could not get trapped by the robot. In addition, an observer holding a wireless emergency stop button was present at all times and responsible for stopping the robot immediately, should any unforeseen hazardous situation arise.

One objective of the study was to assess the three different levels of exchange of explicit information display versions in terms of user performance. This was done by observing the extent to which the displays enabled users to improve task speed depending on the display used. Additionally, the study aimed to evaluate how attractive the display could be in facilitating longer work durations. Another objective was to assess the subjective experience that each condition elicited in users during cooperation with a physical robot. By conducting the study, we aimed to understand the strengths and weaknesses of each level and determine whether greater control over the situation, combined with the level perceived as providing better SA, could lead to an improved UX. As highlighted by San Martin et al. (2024), providing greater control during a task is expected to improve both the UX and performance, particularly in HRC tasks.

For a live demonstration of the designs in a real environment and the robot’s movements, please refer to the following video^6^.

### 4.1 Participants

We decided on using a sample size of 24 participants, relying on research (Nielsen, 2000; Alroobaea and Mayhew, 2014) indicating that for a quantitative/qualitative user study, a sample size of 20 participants is sufficient. Based on this value, we selected 24 participants to ensure a fully counterbalanced design. We recruited 24 participants (13 men and 11 women) with age ranging from 22 to 51 (M=29,SD=5.54) who volunteered to take part in the study. The study was approved by the Institutional Review Board, Tekniker Ethics Committee (TEC) (IRB202300001). Twenty users had previous experience with AR technologies: 11 had previously tried the HoloLens, while the others had previous experience using AR-based games, such as Pokémon Go or decorating apps. The remaining four participants had no prior experience using AR. Nineteen participants had previous experience working with industrial collaborative robots: nine participants had experience programming the robots, six had previously worked with them in industrial tasks such as assembly tasks, and four had both programmed and collaborated with them. The remaining five participants had no prior experience working with robots.

### 4.2 Experimental task

We designed a three-condition, within-subject repeated-measures study to compare the performance, UX, and willingness to work obtained when cooperating with a robot by using each of the three different levels of exchange of explicit information displays. A cooperative task was devised to be completed in each condition. The order in which conditions were presented to the participants was fully counterbalanced, within-subjects, and in each case, the user had to communicate with the robot at least three times.

**FIGURE 4:**
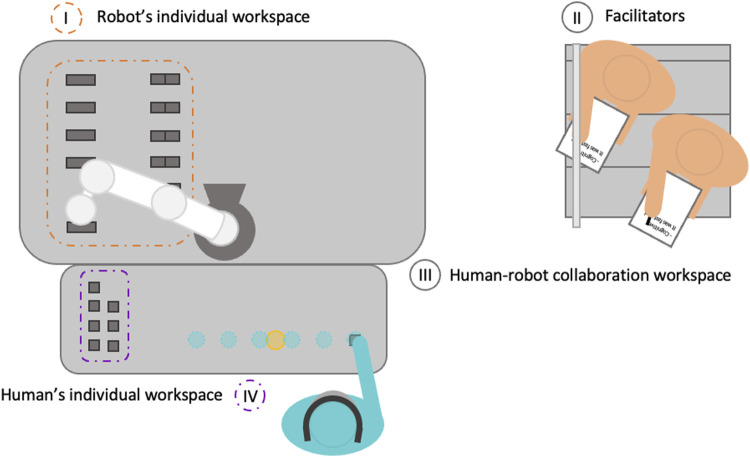
Scenario of the user study. The first step to set up the scenario is placing the calibration yellow holographic sphere in the table in front of the user, so the free spaces appear correctly and is reachable for the user. The bridges and triangles are in the robot’s individual workspace (I). Every time users need any of these pieces, the robot will move it closer to the robot, to the shared space, so users can pick them up (III). The users have next to them the pillars, so they directly can use them to place in the free spaces (IV). During each condition, the experimenters (II) took notes about how the participant performed the task.

The experimental HRC task was set up in an open space of an industrial facility, where noise from machinery that was operating in the same facility could be heard in the background. This choice of environment was representative of an actual industrial facility in which workers (target users of our solution) and robots typically cooperate in real production scenarios. An assembly task was selected, which is representative of tasks commonly found in industrial production (Colim et al., 2020; Gattullo et al., 2020). More specifically, the selected task required the robot to move the bridge and triangle pieces close to the users so that they could construct a building in cooperation with the robot (see Figure 4). The simplicity of this task mirrors the breakdown of complex industrial tasks into smaller, manageable subtasks, as recommended in manufacturing practices (Womack et al., 1990). A real-world example of this approach can be seen in the HRC task presented by ABB (ABB, 2019).

To evaluate the three different levels of exchange of explicit information displays, we wanted a task in which the user and the robot had to occupy the same shared space during the whole execution of the cooperative task.

For that, we devised a task for participants and the robot to cooperate in. The task consisted of creating a building in cooperation with the robot by placing pillars, bridges, and triangles in the free spaces, depicted with blue cylindrical holograms. The user was located in front of the robot, where they could observe the free spaces in the table. The user had to look at the desired piece until the system identified the user’s desire, which was immediately notified at least with a sound (see Section 3.3 for design details). Then, the user could pick up the piece and place it in the desired free space. To block the free space, they had to look at the sphere of the free space for 2 s (explained in Section 3.2). To inform the user that the piece was already blocked in the desired space, the system produced a sound, the red sphere of the free space disappeared, and the space was turned green. When users wanted a piece from the robot, they had to follow the same process: they had to look at the piece and, when identified, the robot would move the piece closer to the user. In case of the C3 design, the user would have to confirm the action before the robot started moving, providing with more information to the robot and whole control to the users.

The task was the same for the three conditions, but the amount of information that was exchanged between the robot and user, and the way in which this affected the flow of the collaboration, changed depending on the condition, as explained in Section 3.3.

Users had to place at least three pillars, two bridges, and a triangle, but they were free to place more pieces if desired. In this way, we could analyze if the willingness and engagement in the task could vary depending on the amount of information provided to users.

### 4.3 Experimental procedure

Each participant read and signed a consent form and filled out a demographic questionnaire. Then, the users were given the HMD with the calibration app so that the eye-tracking system fitted the users’ eyes. After that, the users were given a written description of the task, followed by a more detailed explanation of the task and conditions with the use of a video. Having received the description of the task, every participant received a training session, where they had the chance to execute the task using the different levels of exchange of explicit information displays. In the training session, users had to place two pillars, a bridge above them, and then a triangle. This was repeated with each condition in order to understand how each condition worked. Users carried out the training session for at least 10 min and were given the opportunity to ask any clarification questions they needed.

**FIGURE 5:**
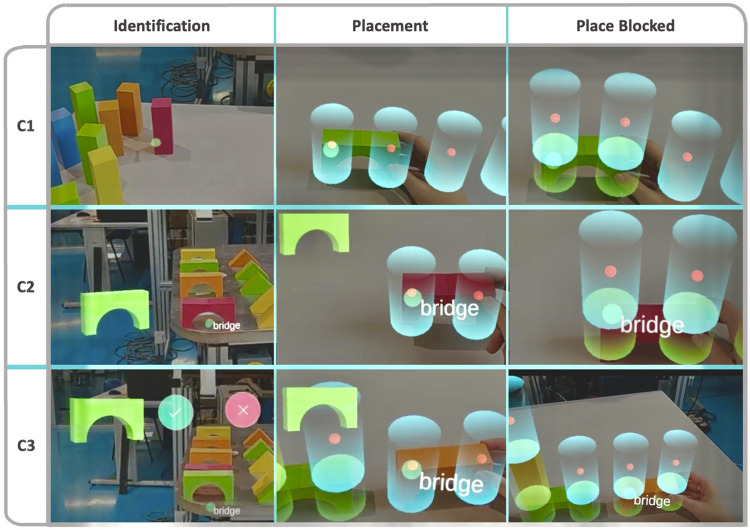
The image shows what the users can see depending on the condition used in each different moment. The first column shows the identification process, where in C1, only audio is displayed; in C2, audio + visual hologram is displayed; and in C3, the same audio and visual holograms as in C2 are displayed + *accept/decline* hologram. In the next two columns, the placement process is depicted, where in C2 and C3, it can be seen that the identification hologram disappears once the place is blocked.

After the training session, the rules of the game were explained to the users so that all users followed them. In the 0 level (over the table), it was only possible to place bridges (which occupied two free spaces) or pillars (one space). Above a bridge, two pillars could be placed; above two pillars, a bridge could be placed; and above a pillar, another pillar could be placed. The triangles (two spaces) could only be placed above a bridge, and nothing else could be constructed above it. Once the rules were explained, the scenario was prepared for the study. To do so, first, one of the facilitators would launch the corresponding condition app. When it was launched, the calibration yellow sphere would appear in front of the facilitator, and it was placed in the table in front of the user, as shown in Figure 4. Then, the next button was pressed; the calibration yellow sphere disappeared; and the free spaces, blue cylinders, would then appear in front of the user. Once the spaces appeared, the glasses were turned back to the user so that they could start with the task. Figure 5 shows how the users interacted with the robot and the pieces and what they saw trough the HoloLens over the three different conditions.

### 4.4 Data collection and analysis

In all conditions, various sets of quantitative data were collected according to selected metrics. One of those metrics was the time taken from the placement of a piece to the next piece placement (performance metric), which was recorded. Additionally, the time needed since the piece was predicted until the piece was placed was also recorded. Finally, the number of pieces placed in each condition was also gathered.

After each condition, the participants responded to three post-task questionnaires: a single ease question (SEQ) (Sauro, 2012) questionnaire, a raw NASA-TLX (RTLX) questionnaire (Hart, 2006), which extended with the category “irritability” (Haas and Edworthy, 1996), and a 3-item version of the situation awareness rating technique (Taylor, 2011); this version is often referred to as 3D SART. After the study, a semi-structured interview was followed. We aimed to analyze aspects such as the following: *how well did they understand the environment? What type of information was perceived in each condition? The overall experience they had in each condition*, and *any other design improvements they suggested*. The interview was enhanced by discussing the participant’s responses provided in the extended RTLX questionnaire and answers given in the 3D SART questionnaires regarding how well the environment was understood. The interviews were recorded and subsequently transcribed for further analysis. The questionnaires were also transcribed and analyzed using the methodology of affinity diagramming, as described by Lucero (2015). For completeness, we briefly summarize the approach. We first created post-it notes that were formatted as follows:

• Top left corner: user number.
• Top left right: possible conditions (C1, C2, and C3).
• Middle: user’s comment.
• Bottom right corner: number of the post-it.

Once all of the comments were transcribed onto the post-it notes, the team read all the post-its individually. Once the first read was done, the team started to discuss the potential clustering for the comments found in the post-its. This iterative process was repeated multiple times, ensuring that all data were correctly interpreted and clustered.

During discussions, the team worked collaboratively to categorize the post-it notes into broader classes and subclasses. The classes were further categorized by specifying whether the comments were positive or negative. When a comment fit in more than one class, it was duplicated (duplicating the post-it number) and placed in all the corresponding categories.

During the execution of the conditions and the post-task questionnaires, two experimenters were involved. One of them observed and interacted with users throughout the entire process, while the other was solely an observer. Both experimenters participated in the development of the data analysis and the creation of the affinity diagram.

## 5 Results

In this section, we present and analyze a comprehensive set of both quantitative and qualitative data. Our approach involves synthesizing insights from these two data types to paint a thorough and nuanced picture of the results obtained. The quantitative data involve pure quantitative data, while qualitative data are divided into two subsections: *quantifying qualitative data*, which involves the data acquired from the questionnaires, and *qualitative data*, which are the data obtained from the semi-structured interview.

For the data analysis, we opt not to rely on null-hypothesis significance testing (NHST). Instead, we embrace using effect size (ES) estimation techniques along with 95% confidence intervals (CI). For the ES evaluation, Cohen’s d (|d|) measure was used as the sample size was n≥20 , and all data met the normality assumption based on Shapiro and Wilk (1965) test ( p>0.05 across all conditions and variables). Cohen’s d is a standardized measure of ES that quantifies the difference between two group means in terms of standard deviations (SD), providing a more informative basis for evidence accumulation. The interpretation of Cohen’s d follows the next established thresholds: a |d| value of 0.2 or higher, but smaller than 0.5 means a small ES, which indicates a fine effect between groups that may still be of interest; a |d| value of 0.5 or higher and smaller than 0.8 means a medium ES, indicating a noticeable and potentially meaningful effect between groups; and a |d| value of 0.8 or greater means a large ES, reflecting a strong effect between the compared groups. As the value is based on comparing two groups (e.g., G1 vs. G2), the d value can be positive or negative. A positive value indicates that G1 has a higher mean value than G2, whereas a negative value indicates that G2 has a higher mean value than G1. These conventions, established by Cohen (1988) and Cohen (1992), are widely used to assess the magnitude of observed effects in research. Additionally, the use of 95% CI, which provides a range of values within which the true ES is likely to be contained, offers a clearer picture of the precision and reliability of the estimated effect. CIs enhance the interpretation of results by providing a context for the possible size of the effect. With this analysis, readers can extract their own critical conclusions, as currently recommended for user studies in disciplines of HRI (Cumming, 2014; Dragicevic, 2016).

### 5.1 Observed quantitative results

**FIGURE 6:**
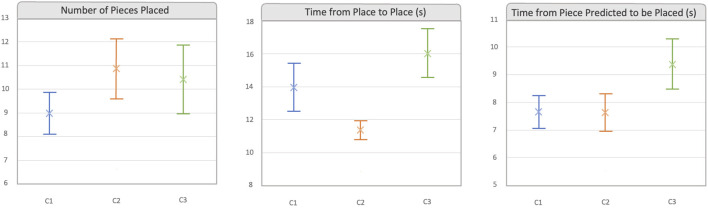
This image shows the mean and 95% of CI for each quantitative data gathered. *Number of pieces placed*: mean number of pieces placed in each condition. *Time from place to place*: mean time (seconds) spent from the placement of a piece to the placement of next piece. *Time from piece predicted to be placed*: mean time spent since the piece is predicted until the user places it in a free space. *C1*: lowest level of explicit information display. *C2*: middle level of explicit information display. *C3*: highest level of explicit information display.

Figure 6 shows all the quantitative data gathered during the experiments. The first graph shows the mean *number of pieces placed* per condition. As explained before in Section 4.2, the participants were required to place at least three pillars, two bridges, and a triangle; however, they could place as many pieces as wanted. If we take a look at the first (left) graph, it shows that there was almost no difference in mean values and CIs of the number of pieces placed between C2 and C3 conditions (C2: 10.875, $CI_{95\%}$ [9.62, 12.13], C3: 10.42, $CI_{95\%}$ [8.96, 11.86]), where participants were provided with more information about the situation. This is also confirmed with the Cohen’s d value (see Table 1), where we can see that the |d| value was below 0.2, meaning that there is a negligible difference. However, we can see a medium effect in the C1 condition (9.0, $CI_{95\%}$ [8.125, 9.87]) compared to that in the C2 (d=−0.693) and C3 conditions (d=−0.501) , where users were provided with less information. This means that users noticeably placed less pieces in C1 compared to other two conditions. In terms of the time elapsed between the consecutive placement of pieces, as shown in the middle graph of Figure 6 and supported by the d values, the mean time (in seconds) for C2 was clearly lower than in the other two conditions (11.398 s, $CI_{95\%}$ [10.83, 11.92]). In both comparisons, a clear large ES was observed with both |d| values over 0.8. Additionally, C3 is higher in this aspect compared to the other two conditions (16.05 s, $CI_{95\%}$ [14.6, 17.504]), with a medium ES compared to C1 and large compared to C2. However, it has to be pointed out that in the C3 condition, users had to confirm the action of the robot, when they requested a bridge or triangle, by looking at the *accept* button for 2 s (explained in Section 3.3, 3.4). This aspect is reflected in the last variable gathered: *time elapsed since the system identified which piece the user wanted, until that piece was placed*. The mean time (in seconds) was largely higher with the C3 condition (9.37 s, $CI_{95\%}$ [8.476, 10.277]), with a mean time of 1.77 s higher than in the other two conditions, which is also reflected with |d| values over 0.8. This is the period of time when users had to accept/reject the action.

**TABLE 1:** Numerical Cohen’s d values for the data presented in Figure 6.

Condition	Number of pieces placed	Time from place to place (s)	Time from piece predicted to be placed (s)
C1 vs. C2	−0.693	0.938	0.009
C1 vs. C3	−0.501	−0.572	−0.916
C2 vs. C3	0.135	−1.695	−0.876

All numerical values related to the quantitative results are presented in the Supplementary Material.

### 5.2 Quantifying qualitative results

**FIGURE 7:**
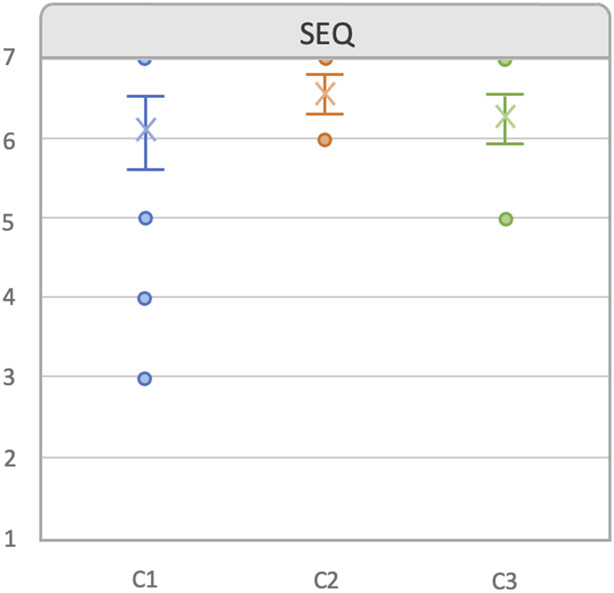
Responses to the Single Ease Question (SEQ) post-task questionnaire, presented by condition. Error bars represent 95% confidence intervals.

Figure 7 shows the answers provided by users in the SEQ questionnaire, rated in a 7-scale Likert scale, on *how easy it was to perform the task in each condition* (Sauro, 2012). Although there was overlap in all CIs, it can be observed that the mean of C2 condition (6.85, $CI_{95\%}$ [5.63, 6.54]) was slightly bigger than in the C3 (6.54, $CI_{95\%}$ [6.3, 6.77]) and C1 (6.25, $CI_{95\%}$ [5.93, 6.56]) conditions. It is also observable that in C1, the CI was bigger than in other conditions and that there were more out-bounding points than in the other two conditions, with almost all of them being in lower values. Lower values mean that the task was not that easy to perform with this condition. The advantage of C2 over C1 an C3 is further corroborated by the ES analysis. The comparison between C1 and C2 gave a medium ES (d=−0.506) , while C2 vs. C3 resulted in a small effect size (d=0.417) . In both comparisons, C2 was perceived as making the task easier to perform.

**FIGURE 8:**
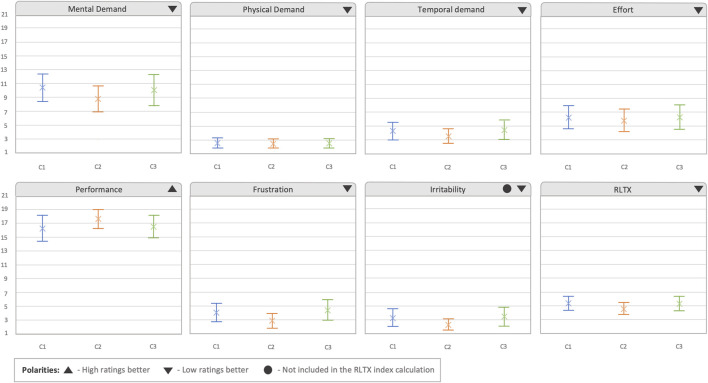
Results obtained from the extended raw NASA-TLX questionnaire, presented by condition. The irritability category was added as an extension to the questionnaire, and therefore, it was not used to calculate the RTLX index. The graphs show average values per condition. Error bars represent 95% confidence intervals.

In the raw extended NASA-TLX (RTLX) analysis, the *irritability* term was not used to calculate the RTLX index. We added this category since we added the confirmation prompt, which could make the user go slower and, hence, we wanted to analyze if there could be an effect in the experience perceived by the user. As shown in Figure 8, the differences in mean values observed between the six categories were small. However, it can be perceived that in *mental demand*, *temporal demand*, *frustration*, and *irritability*, the C2 condition appeared to have the CI in lower ratings than that in the other two conditions and higher values in *performance*, which also resulted in slightly lower ratings in the RTLX. These differences are further confirmed when taking a look to the ES evaluation (see Table 2). Small ESs were observed in *mental demand*, *performance*, *frustration*, *irritability*, and *RTLX*. In all these aspects, the mean value in C2 was slightly smaller than that in the other conditions, expect for *performance*, where C2 was slightly higher. Additionally, in the *temporal demand*, another small ES was also observed compared to C1, with C2 being perceived as being slightly less demanding.

**TABLE 2:** Evaluation of ES of raw NASA-TLX based on Cohen’s d calculation.

Condition	Mental demand	Physical demand	Temporal demand	Effort
C1 vs. C2	0.335	0.050	0.263	0.113
C1 vs. C3	0.071	0.025	−0.065	0.0
C2 vs. C3	−0.242	−0.026	−0.143	−0.111

Condition	Performance	Frustration	Irritability	RTLX
C1 vs. C2	−0.354	0.402	0.381	0.345
C1 vs. C3	−0.068	−0.106	−0.051	0.011
C2 vs. C3	0.300	−0.491	−0.423	−0.325

**FIGURE 9:**
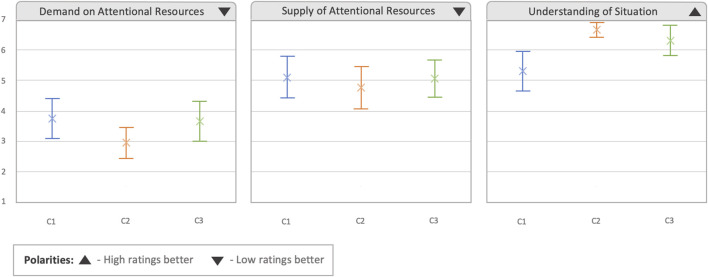
Results obtained from the 3D SART questionnaire. Error bars represent 95% confidence intervals.

The last questionnaire performed after each condition was the 3D SART, which had the aim of analyzing the situation awareness obtained in each condition. As can be observed in Figure 9, in the first two categories regarding the *demand and supply of intentional resources* (how much information does the system demand from the user, and how much attention have the users provided to the system), there was an overlap in the CIs of the different conditions, although the mean and CIs seemed to be smaller in the C2 condition (demand: 2.958, $CI_{95\%}$ [2.45, 3.465], supply: 4.79, $CI_{95\%}$ [4.104, 5.479]) compared to that in the other two conditions. However, in the *understanding of situation* category, the C2 condition was clearly higher in mean value (6.667, $CI_{95\%}$ [6.44, 6.89]) compared to the C1 condition (5.33, $CI_{95\%}$ )[4.68, 5.9587])]), obtaining a confident interval, which did not overlap with the C1 condition. The C3 condition also showed a higher mean value (6.33, $CI_{95\%}$ [5.83, 6.83]) compared to the C1 case, where a lower amount of information was provided to users. The differences observed in both aspects are further confirmed with the Cohen’s d values (see Table 3). In the *demand and supply of intentional resources*, a medium ES was observed in respect to C1, and small one in respect to C3, perceiving C2 as less demanding. Additionally, analyzing the *understanding of situation*, we can observe that C1 was observed as the worst condition for understanding the situation, with respect to other two conditions, with a large effect size compared to C2 and medium effect size compared to C3.

**TABLE 3:** Evaluation of ES of the 3D SART questionnaire based on Cohen’s d calculation.

Condition	Demand on attentional resources	Supply of attentional resources	Understanding of situation
C1 vs. C2	0.538	0.196	−1.091
C1 vs. C3	0.051	0.026	−0.690
C2 vs. C3	−0.490	−0.179	0.346

The numerical values regarding NASA-TLX and 3D SART are added in the Supplementary Material.

### 5.3 Qualitative results

**FIGURE 10:**
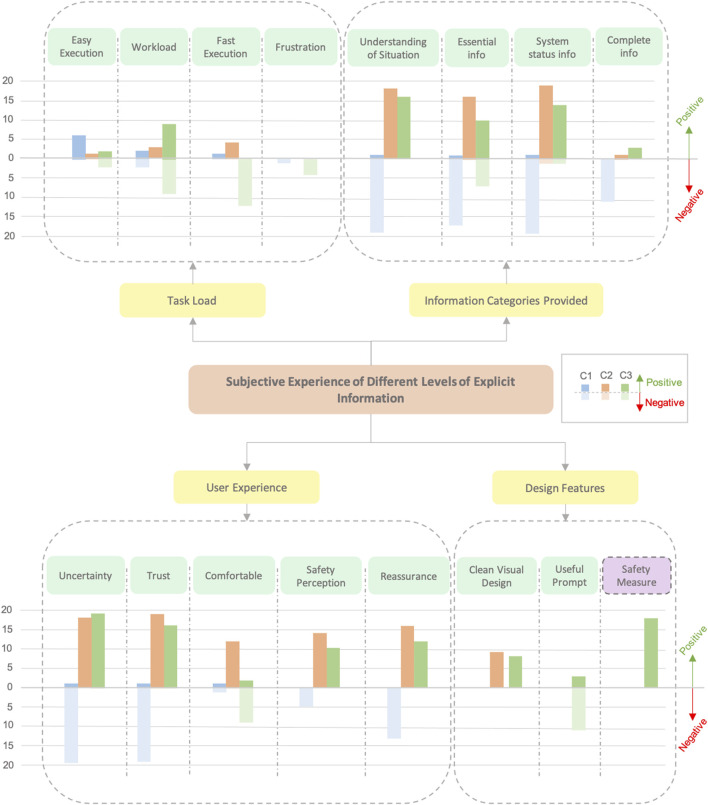
This is the affinity diagram obtained from the analysis of subjective statements gathered from participants during semi-structured interviews. Each branch category in the diagram includes graphs indicating the number of participants who offered positive (up on the scale) or negative (down on the scale) comments related to that category concerning a specific condition in the study.

**FIGURE 11:**
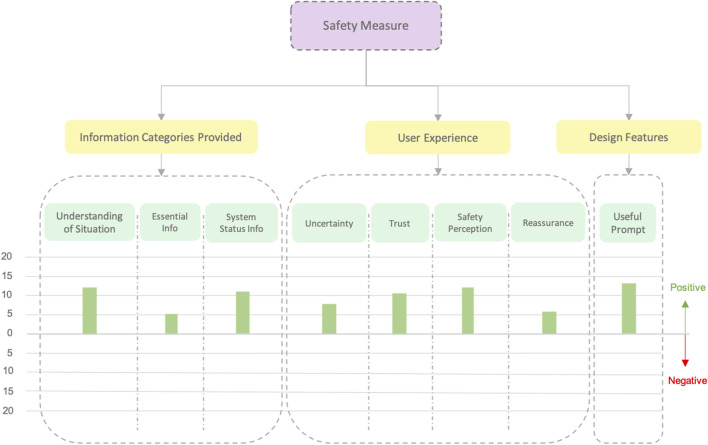
Affinity diagram obtained from the comments made by users about the possibility of using the confirmation prompt in dangerous situations. In this case, we only analyze C3 since it was the category providing this functionality.

Once the affinity diagram was obtained following the steps explained by Lucero (2015) (see Section 4.4), the affinity diagram was transcribed digitally using Excel. The data entered in Excel were transformed into graphs to enhance the readability of the affinity diagram (see Figures 10, 11). Both graphs comprised comments and responses from participants to the facilitator’s questions. The first resulting graph, presented in Figure 10, led to the categorization of four main groups: *task load*, *information categories provided*, *user experience*, and *design features*. These main groups were further delineated based on various characteristics, as illustrated in Figure 10. The transcription of all participant comments produced 139 post-it notes, which were most successfully organized in the four branch categories just mentioned, which contained 16 leaf categories in total. The following is an outline of each leaf, from left to right:

• **Easy execution.** Comments on how easy it was to perform the task with the information provided by each condition.
• **Workload.** The workload perceived in each condition while executing the task.
• **Fast execution.** How fast the users perceived that they were performing the task.
• **Frustration.** Comments about feeling frustrated during the execution of the task.
• **Understanding of situation.** Comments about being able to correctly understand what was going on in the system.
• **Essential information.** Comments about conditions providing at least the essential information to know that the system was working properly.
• **System status information.** The user could understand at which point the system was, if the system has already identified something, if the robot will move, and so on.
• **Complete information.** Comments on how complete the information received was.
• **Uncertainty.** Experiencing uncertainty of what was happening while performing the task.
• **Trust.** Comments about users trusting the system.
• **Comfortable.** Comments about feeling good and comfortable while they were performing the task.
• **Safety perception.** Experiencing the feeling of safety while cooperating with the robot.
• **Reassurance.** Comments about feeling reassured that the system was working properly.
• **Clean visual design.** The visual design did not clutter or disturb the task.
• **Useful prompt.** Comments regarding whether the confirmation prompt of C3 was useful for cooperating with the robot.
• **Safety measure.** Comments about if the prompt of C3 was an important tool for safety.

During the semi-structured interview, many participants pointed out that the prompt or confirmation request (accept/decline button) by the robot in C3 was good as a safety measure. Hence, we asked some more questions to participants about whether they thought it could be useful for dangerous situations and how they would feel using this prompt in such situations. Therefore, we performed a zoom-in into the *safety measure category* and obtained another affinity diagram and, hence, a second graph regarding how C3 could affect in dangerous scenarios (See Figure 11). As can be observed, regarding dangerous situations, all comments were positive.

## 6 Discussion

The research we present in this paper is exploratory and, hence, there is scarce research in the literature that we can use as comparison to our work.

Results obtained in this study show that the amount of explicit information exchanged provided through an MR interface do directly affect the UX, task engagement, and performance during HRC tasks. Specifically, conditions C2 and C3, which offer progressively more exchange of information to users, outperform condition C1 in most categories, except for the task load aspect.

### 6.1 The role of the amount of information in enhancing performance and task engagement

One of the key findings is that providing higher levels of explicit information not only improves task performance, consistent with previous research (De Franco et al., 2019; Riley et al., 2012), but also increases users’ engagement.

In conditions with auditory and visual feedback (C2 and C3), users placed more blocks during the task than with minimal information (C1). The importance of information is also present in task performance, where C2 showed the best task rhythm. However, C3 showed the slowest rhythm, but it is attributed to the confirmatory step where users lost at least 2 s every time they asked for one of the pieces of the robot (explained in Section 5.1). This inconvenience was confirmed by users’ comments such as *“The prompt confirmation slowed me down”* (P02). Interestingly, six out of 24 users noted that C1 was perceived as more simple to understand and hence of *easy execution* (*“This is faster and easier to use”* (P19)) and *fast execution* (*“you go faster”* (P17)). This suggests that providing minimal information can sometimes reduce cognitive load, making the interaction feel lighter. Nevertheless, this perceived simplicity did not translate into better performance as users performed worse in C1 than in C2. The quantitative results align with comments from other users who mentioned the need to adjust their head and eye gaze to guide the system toward correct identification, potentially leading to wasted time.

Results indicate that C2 provided an optimal balance between situational awareness and cognitive load. This is reflected in the UX, where all comments recorded were positive, and in the NASA-TLX, where aspects such as *mental demand*, *performance*, *frustration*, and *irritability* were rated slightly better (with small ES) compared to the other two conditions. Additionally, users rated C2 as the easiest condition to use on the SEQ questionnaire, with a medium ES compared to C1 and small in respect to C3. The clarity of visual feedback in C2 allowed users to understand the robot’s actions without adding excessive workload.

### 6.2 Building trust through transparency without decreasing UX

Results suggest that the *trust* users experienced was tightly connected to how well they could understand the robot’s intentions and actions. C2 and C3 received higher and more positive statements in terms of *trust* as these conditions offered greater transparency in the interaction. This transparency is evident in the *information categories provided* branch, where comments regarding C2 and C3 were positive, indicating that both displays were good tools to *understand the situation* (18/24 for C2 and 16/24 for C3), *provide essential information* (16/24 for C2 and 10/24 for C3), and *obtain accurate system status information* (19/24 for C2 and 14/24 for C3).

The visual feedback in C2 and C3 conditions not only made users feel that the system was correct at interpreting their intentions (making them feel *reassured*) but also improved their *sense of safety* during cooperation. As stated by users: *“Feedback allows to check system’s proper working”* (P01), *“Hologram information makes me feel safe and reliable”* (P05), and *“I need to have visual information to be sure the system is understanding me and to feel safe”* (P18). These aspects led to a scenario where the feelings of *uncertainty* were reduced without cluttering the task environment (*“The visual cues are not cluttering”* (P01)).

However, although the additional information provided by users in C3 was particularly effective in *providing complete information*, with statements such as: *“The information is complete”* (P14) and *“It provides the most complete information”* (P21); it also caused some drawbacks. Nine out of 24 users reported that they felt more mental effort was required in this condition despite small differences that were recorded in the NASA-TLX results. Another negative aspect of the UX related to the *comfort* aspect was where several users mentioned not feeling comfortable (*“I feel uncomfortable having to use the prompt”* (P02)). These factors, added to the time lost, could explain why 10 participants felt that the confirmatory prompt was not necessary for the task at hand as it made the interaction more cumbersome without adding any perceived value for simpler tasks.

In contrast to the trust-enhancing effects of C2 and C3, the lack of clarity in C1 regarding what the system was identifying led to a sense of *mistrust* among participants (17 out of 24). C1 created a scenario of *uncertainty*, where users *did not feel safe*, resulting in a lack of trust when cooperating with the robot (*“With the auditive cue only I don’t feel safe, because I do not trust the system”* (P14); *“I cannot be sure if the system is working properly”* (P23)). This suggests that in HRC scenarios, users need to have complete information about what exactly the robot is identifying, which is not satisfied only by the use of auditive cues. Comments such as *“Without the visual feedback, you must assume that the system is identifying well”* (P01), *“I need more information”* (P19), or *“With audio only I cannot be sure if the system is working properly”* (P21) reinforce this idea of needing explicit visual feedback.

### 6.3 Balancing safety and efficiency: implications for design in HRC tasks

Although C2 was generally preferred for its balance of information and efficiency, the explicit information and control provided in C3 was considered highly valuable in safety scenarios. Participants believed that having the ability to confirm or reject actions, and knowing exactly when the robot will start moving, would be crucial when working with robots in potentially hazardous environments, turning the perceived inconvenience of slower task progression into a benefit for safety. Eighteen out of the 24 participants made comments such as: *“Although you go slow, you feel more safe because you have whole control over the situation”* (P15). This led to general positive feedback comments about the use of this condition as safety measures (see Figure 11).

The difference in UXs and preferences for the type of feedback (8/24 for C2 *“I prefer C2 condition, it gives more information”* (P05), 03/24 for C3 *“This is the best condition”* (P20), and 0/24 for C1 *“This is the worst condition”* (P18)) underscore the importance of adaptable HRC MR systems. Such systems would allow for different levels of SA and control based on the task’s safety requirements, while always providing explicit information about the interaction.

## 7 Design and implementation limitations

The user study reported here does not provide data to estimate the performance and acceptance with designs other than the ones presented and evaluated here. Thus, research groups investigating related questions might want to explore and evaluate this design space further. For example, P03 pointed out that as a future step, it could light-up the possible places where the identified piece could be placed. P07, P08, P10, P11, and P12 also mentioned that adding sound when the confirmation prompt is accepted/denied could be interesting.

Another limitation comes from constraints in the current technology to recreate an ecologically valid visual field. Although HoloLens 2 has a wider field of view (54°) than its preceding version (34°), it is still much narrower than the field of view of human vision. However, we used sound that can be heard regardless of where the user is looking at and a fixed hologram for identification, so users could observe it, although they were turning the head. The only things that were not seen from any point were free spaces that were fixed in the table.

Regarding the performance capacity of the selected HMD, a few limitations determined the time we set for the object identification. HoloLens 2^7^ has a Qualcomm Snapdragon 850 Compute Platform CPU with 4-GB LPDDR4x system DRAM of memory. In terms of software, it already has functionalities built in for human understanding such as hand tracking, eye tracking, and voice command recognition. These functions consume processing resources of the HMD, and we added to it real-time streaming of data, image included, which was slowing down the system performance; this is the reason we used a minimum identification time of 600 ms. In the worst observed scenario, it was prolonged to 800 ms since, while more places where blocked, new places appeared, increasing the rendering of the HMD and, hence, delaying the data exchange.

The resolution of the last two constraints discussed is dependent on the advancement of the technology used in future HMD devices.

## 8 Conclusion and future work

Motivated by the increasing use of cobots, and having in mind how users communicate and collaborate, we wanted to develop a technique capable of identifying what users wanted at each stage in the context of a cooperative task. We developed three different display designs with which to provide users with three incremental levels of explicit information exchanged regarding the ongoing cooperation with the robot. The information displayed was intended for the human user to be aware of the situation at each point in time and throughout the entire cooperation with the robot. In cooperative scenarios in which both robot and human workers share the same space, the robot must understand the user and vice-versa. The goal was to make the robot able to properly identify the piece that the user wants and, at the same time, transmit to the user the information that helps them understand what is happening and what is about to happen, without adding any unnecessary exchange of information between both.

Regarding the performance of the participants in the study, in terms of pace task execution, the C2 condition led to better results. In conditions C2 and C3, where participants had additional information about the ongoing situation, they remained more engaged in the cooperative task. In those conditions, they placed more pieces in the building they were constructing together with the robot. In other words, when the information was scarce to permit staying well-aware of the situation, participants chose not to extend the cooperation much beyond what was strictly necessary.

The C2 condition, where the robot was allowed to execute actions without the need for the user to validate each one of them beforehand, resulted in the most effective condition in terms of performance and UX, as well as being the preferred one. The triangulation of quantitative and qualitative results from the user study supports the notion that more complete awareness information conveyed reassurance for a proper bidirectional communication. Such awareness of the situation allowed users to identify that the robot was understanding their wishes correctly and to be aware of the status of the robot at all times. Even though users were not given the whole control over the robot´s action, this C2 condition has been shown to be the best to improve the UX of users. In addition, the information provided to users has been shown to not clutter the task and be a helpful tool, although a minority of participants preferred the simplicity of single-modality displays.

Changing contextual and environmental conditions in the cooperative scenario (including lighting and noise) together with the diversity of profiles of human workers (with different preferences and perceptual capacities) may require the reliance of SA solely on the information from one of the sensory channels. In that sense, the study showed that audio-only information was easier to understand and fast to execute, although it did not give reassurance to users about what was happening exactly. Hence, for a more comprehensive and descriptive situation, visual information has shown higher strengths for understanding the ongoing situation.

A last interesting aspect to analyze is the role of the user as the validator of every action of the robot (condition C3). If we analyze both quantitative and qualitative data, it can be observed that some aspects are negatively accepted due to the delay created by the confirmation step. However, the qualitative results did also show that this simple step could be really helpful in more dangerous situations, where users could feel a higher degree of danger. They believed that, in such scenarios, the prompt would be really useful and, in such cases, almost all negative aspects observed in the UX analysis would disappear.

One of the key aspects of this study is the synergy between the robot’s cognitive ability to reason and respond and the user’s enhanced situational awareness. This dual advancement fosters an environment of seamless and effective human–robot collaboration, where the robot and user mutually enhance each other’s capabilities. By creating systems where robots understand users’ needs and users remain aware of the robot’s actions, this study highlights a fundamental step toward the future of collaborative systems.

With these conclusions in mind, an interesting step in future would be to analyze the effect of complete control of the human over the robot’s actions in scenarios and tasks that users perceived as hazardous. The design of such a task should be perceived as dangerous by users, but safety would be maintained throughout the whole cooperative task.

Finally, future work could explore the integration of other sensory cues, such as facial expressions or voice recognition, to create smoother and more personalized interactions. It would also be interesting to study how these additional cues could enhance situational awareness across different sensory modalities, particularly in scenarios in which a single channel might be limiting due to occlusions resulting from, e.g., changing environmental conditions.

To wrap up, although an intermediate level of SA is suitable for most HRC scenarios, careful attention must be paid to factors such as risk, user preferences, and the need to design a system that ensures effective bidirectional communication.

## Data Availability

The raw data supporting the conclusions of this article will be made available by the authors, without undue reservation.

## Footnotes

1
https://github.com/microsoft/MixedReality-WebRTC

2
https://github.com/ultralytics/yolov5

3
https://moveit.ros.org

4
https://learn.microsoft.com/en-us/windows/mixed-reality/design/eye-tracking

5
https://app.roboflow.com

6
https://drive.google.com/file/d/179_pj3SistuV59--16dZbhNGxaD4PRGc/view?usp=sharing

7
https://docs.microsoft.com/en-us/hololens/hololens2-hardware
