# Supplementary material for: An analysis of the role of different levels of exchange of explicit information in human–robot cooperation
Source: Front Robot AI. 2025 Feb 10;12:1511619. doi: 10.3389/frobt.2025.1511619 (PMC11848069; doi:10.3389/frobt.2025.1511619)
Supplement: Supplementary file 1 [file DataSheet1.pdf]

Supplementary Material

1 NUMERIAL VALUES OF QUANTITATIVE RESULTS

Table S1. Numerical values of means and confidence intervals corresponding to quantitative data for each condition. Values represent 95% confidence intervals.

Condition	Number of Pieces Placed	Time from Place to Place (s)	Time from Piece Predicted to be Placed (s)
**C1**	9.0 [8.125, 9.87]	13.98 [12.53, 15.43]	7.64 [7.064, 8.22]
**C2**	10.875 [9.62, 12.13]	11.398 [10.83, 11.962]	7.63 [6.95, 8.3]
**C3**	10.42 [8.96, 11.86]	16.05 [14.6, 17.504]	9.37 [8.47, 10.277]

2 NUMERICAL VALUES OF NASA-TLX AND 3D SART

Table S2. Results from the extended raw NASA-TLX questionnaire: average values and 95% confidence intervals

Condition	Mental Demand	Physical Demand	Temporal Demand	Effort
**C1**	10.458 [8.459, 12.457]	2.541 [1.853, 3.229]	4.25 [3.016, 5.483]	6.25 [4.539, 7.907]
**C2**	8.83 [6.958, 10.708]	2.458 [1.8323, 3.093]	3.5 [2.458, 4.542]	5.79 [4.191, 7.391]
**C3**	10.083 [7.848, 12.318]	2.5 [1.843, 3.156]	4.417 [3.021, 5.812]	6.25 [4.539, 7.96]

Condition	Performance	Frustration	Irritability	RTLX
**C1**	16.29 [14.48, 18.0943]	4.041 [2.704, 5.378]	3.25 [1.988, 4.511]	5.375 [4.383, 6.366]
**C2**	17.69 [16.32, 19.061]	2.83 [1.7803, 3.886]	2.25 [1.465, 3.034]	4.5763 [3.72, 5.432]
**C3**	16.583 [14.966, 18.20]	4.417 [2.924, 5.908]	3.417 [2.0667, 4.766]	5.347 [4.311, 6.382]

Table S3. Numerical values of means and confidence intervals corresponding to the 3D SART questionnaire. Values represent 95% confidence intervals.

Condition	Demand on Attentional Resources	Supply of Attentional Resources	Understanding of Situation
**C1**	3.75<br>[3.09, 4.409]	5.125<br>[4.454, 5.795]	5.33<br>[4.68, 5.987]
**C2**	2.958<br>[2.45, 3.465]	4.79<br>[4.104, 5.479]	6.667<br>[6.44, 6.89]
**C3**	3.66<br>[3.024, 4.309]	5.083<br>[4.47, 5.695]	6.33<br>[5.83, 6.83]
